# A Novel Method for Estimating Knee Angle Using Two Leg-Mounted Gyroscopes for Continuous Monitoring with Mobile Health Devices

**DOI:** 10.3390/s18092759

**Published:** 2018-08-22

**Authors:** Eric Allseits, Kyoung Jae Kim, Christopher Bennett, Robert Gailey, Ignacio Gaunaurd, Vibhor Agrawal

**Affiliations:** 1 Department of Biomedical Engineering, University of Miami, Coral Gables, FL 33146, USA; eallseits0@gmail.com (E.A.); bennett@miami.edu (C.B.); 2 Department of Physical Therapy, Miller School of Medicine, University of Miami, Miami, FL 33136, USA; kjkim@miami.edu (K.J.K.); rgailey@miami.edu (R.G.); 3 Frost School of Music, Music Engineering Technology, University of Miami, Coral Gables, FL 33146, USA; 4 Miami Veterans Affairs Healthcare System, Miami, FL 33125, USA; Ignacio.gaunaurd@va.gov

**Keywords:** inertial measurement unit (IMU), gyroscope, knee angular velocity, knee joint angle, gait analysis, mobile health

## Abstract

Tele-rehabilitation of patients with gait abnormalities could benefit from continuous monitoring of knee joint angle in the home and community. Continuous monitoring with mobile devices can be restricted by the number of body-worn sensors, signal bandwidth, and the complexity of operating algorithms. Therefore, this paper proposes a novel algorithm for estimating knee joint angle using lower limb angular velocity, obtained with only two leg-mounted gyroscopes. This gyroscope only (GO) algorithm calculates knee angle by integrating gyroscope-derived knee angular velocity signal, and thus avoids reliance on noisy accelerometer data. To eliminate drift in gyroscope data, a zero-angle update derived from a characteristic point in the knee angular velocity is applied to every stride. The concurrent validity and construct convergent validity of the GO algorithm was determined with two existing IMU-based algorithms, complementary and Kalman filters, and an optical motion capture system, respectively. Bland–Altman analysis indicated a high-level of agreement between the GO algorithm and other measures of knee angle.

## 1. Introduction

Recent advances in mobile device technology and body-worn inertial measurement unit (IMU) sensors have made continuous monitoring of movement in the home and community possible. Continuous monitoring can play an important role in health care delivery by providing a platform for tele-health and self-management interventions. For example, medical conditions, such as lower limb amputations and walking with a prosthesis, can impair a person’s walking ability and result in a higher risk for stumbles and falls. Long-term rehabilitation programs would benefit from continuous monitoring of lower limb and knee movements to identify changes in biomechanics that can lead to stumbles and falls. For this reason, continuous assessment of knee joint angle in the home and community could lead to novel interventions for “normalizing” knee movement patterns and increasing mobility and safety for a variety of medical conditions.

Knee angle can be measured by a variety of instruments, such as optical motion capture systems, IMU based systems, and electrogoniometers. All three system types measure knee angle as a gross anatomical approximation that simplifies the two distinct joints of the knee into a single joint [1,2]. For example, optical motion capture systems (OMCS) and many IMU systems rely on a Three-Dimensional Gait Analysis (3DGA) paradigm that is generalizable to any motion tracking problem. It consists of determining absolute sensor location in space, using pre-determined sensor-to-body rotations and translations and biomechanical models to specify limb segment orientation and location in space. This paradigm estimates knee angle as the relative difference in the extrapolated shank and thigh orientations. Such over-specification of sensor orientation in three-dimensional space is inappropriate for clinical, continuous monitoring applications [3,4]. Additionally, sensor placement fidelity and calibration are essential in 3DGA applications and become problematic in user driven data acquisition paradigms. Sensor number and bandwidth restrict algorithm complexity in mobile applications. Current practice in IMU knee angle calculation using Kalman filters requires use of multiple sensor types which includes gyroscopes and accelerometers, and sometimes magnetometers. However, gait deviations, walking on a prosthesis, scar and adipose tissue can cause accelerometer data to be corrupted by vibratory noise, especially during stance phase of gait. Excessive noise can result in inaccurate joint angle calculations, especially for the disabled population.

Many studies have attempted to validate IMU derived knee joint rotation in three dimensions [5,6] and have had varying degrees of success. The majority of studies have focused on estimating only the flexion/extension of the knee under the assumption that the knee operates as a simple hinge joint. A commonly used method for determining knee flexion/extension [7,8] exploits the closed chain kinematic constraints of walking and combines accelerometer and gyroscope data using a complementary filter. Since accelerometer data are sensitive to placement variability and can contain significant amounts of Gaussian noise and gait cycle dependent noise from vibratory modes, its application can be problematic for mobile applications. Therefore, in this study, a novel gyroscope only (GO) algorithm is proposed which calculates knee angle from integration of a gyroscope derived knee angular velocity (KAV) signal that avoids reliance on noisy accelerometer data. To eliminate drift in the integral of angular velocity, a zero-angle update (ZAU) derived from a characteristic point in the knee angular velocity is applied to every stride. This point is identified using gait phase knowledge provided by a previously published noise-zero crossing (NZC) gait phase algorithm [3]. The goal of this study was to determine validity of the GO algorithm for calculating knee flexion/extension angles with a low-power IMU system and an iPad.

Validity of the GO algorithm was assessed with two methods: (i) concurrent validity was determined through agreement with a previously published knee angle algorithm [8]; and (ii) construct convergent validity was established with an OMCS. 

## 2. Methods 

### 2.1. Experimental Systems and Design

**Figure 1:**
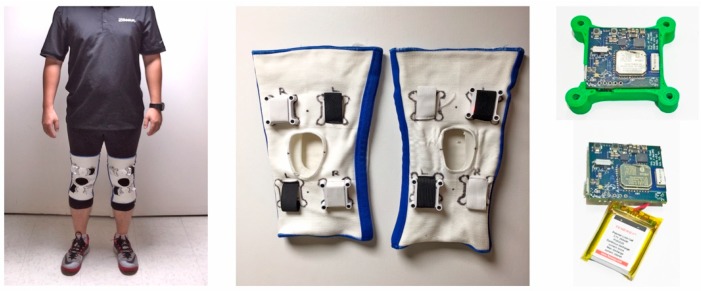
Depiction of MAPR system after donning by a subject (**left**); MAPR IMUs embedded in knee sleeves (**middle**); and circuit board of MAPR IMU (**right**).

Three different experimental systems were used in this study: a Vicon Bonita OMCS (Vicon Motion Systems Ltd., Oxford, UK), a commercial Opal (APDM Wearable Technologies, Portland, OR, USA), and an in-house Multi-Axial Profile Recorder (MAPR) system. The MAPR system (Figure 1) was developed as a low-power hardware IMU sensor designed for continuous monitoring applications in which ease of use and extended battery life are paramount. MAPRs were designed to transmit IMU data in real time to an iPad using a Bluetooth Low Energy wireless transmission protocol to extend battery life and minimize packet loss. 

To do so, the characteristics of the knee angular velocity curve were compared between the MAPR and Vicon systems to justify use of a zero knee angle update point to eliminate integratory drift. The concurrent validity of the GO algorithm was assessed using three different criterion measures. First, peak swing flexion was compared between the Vicon system and the MAPR/GO system. Next, peak swing flexion and RMSE knee angle were compared between the GO algorithm and the established CF algorithm [8]. Since the CF algorithm was validated using a commercially available IMU system that employs a sample–decimate–transmit protocol, it is unknown whether the algorithm would perform as well using data collected via the clinically oriented MAPR system. Thus, this within IMU knee angle comparison was conducted using Opal and MAPR data collected simultaneously. Finally, peak swing flexion and RMSE were compared between the GO algorithm and an Extended Kalman Filter using the MAPR system.

### 2.2. Gyroscope Only (GO) Algorithm

The GO algorithm was designed to compute an accurate estimate of flexion/extension of the knee during gait. Once aligned to the knee joint axis, shank and thigh gyroscope data can be differenced from each other to derive an estimate of KAV and the KAV can be integrated to obtain a knee angle estimate $\Psi_{g}$ using only one sensor type. This signal is corrupted by integratory drift, which is typically accounted for by fusing data from either accelerometers or magnetometers. 

**Figure 2:**
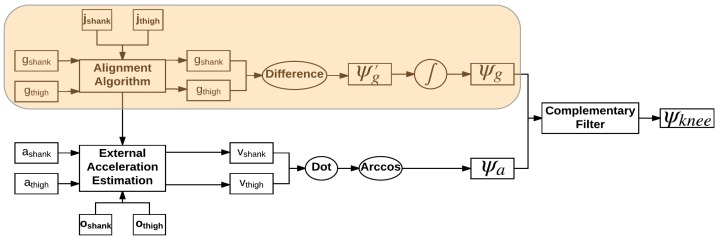
Flowchart of the complimentary filter method to calculate knee joint angle [8]. Alignment algorithm rotates the measured angular velocities ***g*** to be aligned with the joint axis ***j*** (orientation vector of the joint axis in the sensor reference frame). ***a***, acceleration measured by the accelerometer; ***o***, displacement vector of the joint axis in the sensor reference frame; ***v***, acceleration due to gravity; ***Ψ****’*, knee angular velocity; ***Ψ***, knee angle.

In mobile applications where battery life is a system design concern, increasing bandwidth by including a second sensor type is undesirable. Because of the characteristic waveform of lower limb angular velocity curves during gait, several studies describing the physiologic meaning of peaks, troughs, or zero-crossings of the gyroscope signal from sensors placed on feet, shanks, thighs, and the sacrum have been conducted over the years [9,10,11,12,13,14]. Using gait event driven information, it should be possible to produce an estimate of knee angle using only angular velocity of the lower limbs, as depicted in the shaded region of Figure 2, if there exists an easily identifiable point in the KAV signal that corresponds to a minimum in the knee angle and if the minimum point in the knee angle can be used to reset the integral of the KAV to zero once per stride. 

It was demonstrated by the analysis of the KAV that both these conditions are met. The KAV during flexion and extension is a quasiperiodic signal composed of positive and negative regions that correspond to the physiologic motions of flexion and extension of the knee joint. It should have four zero crossings per stride. When rate of change of the knee angle (KAV) goes from positive (flexion) to negative (extension), this corresponds to a minimum in the knee angle, which should occur twice per stride: once between mid-stance and toe-off and once between mid-swing and heel-strike. Resetting initial conditions during integration of IMU signals at characteristic gait events has been successfully employed in the calculation of step length and gait speed [14,15,16]. For example, detection of mid-stance has been used to apply a zero-velocity update to the velocity integral for eliminating the accumulated drift. A similar approach should be effective in calculation of knee angle from the gyroscope signal. If a ZAU point is robustly identifiable in the KAV signal, knee angle can be directly integrated over each gait cycle from the gyroscope derived KAV signal.

**Figure 3:**
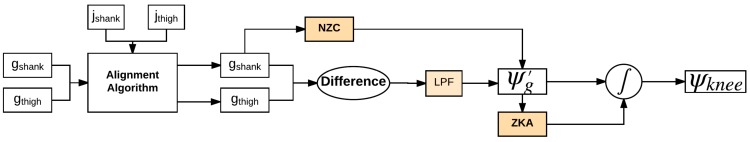
Flowchart of the GO algorithm. *j*, orientation vector of the joint axis in the sensor reference frame; *g*, measured angular velocity; ***Ψ****’*, knee angular velocity; ***Ψ***, knee angle; NZC, Noise-Zero Crossing; LPF, Low Pass Filter; ZKA, Zero Knee Angle.

As shown in Figure 3, the GO algorithm utilizes knowledge of gait events to reliably detect the zero-crossing prior to the start of knee flexion and apply a ZAU derived from a characteristic peak in the knee angular velocity to every stride to compensate for integratory drift. A zero-crossing detection algorithm is in the OFF position until MSt is detected using the shank angular velocity signal [4]. Once MSt is detected, the algorithm waits until the minimum prior to TO is detected and takes the most recent positive to negative zero-crossing of the KAV signal as the point where the knee angle is reset to zero. Once knee angle is set to zero, the zero-crossing detection algorithm switches to OFF and the knee angle is calculated by accumulation of the low pass filtered KAV signal obtained as described previously. A single reset per gait cycle is proposed and supported by analyzing the average of the integral per stride.

### 2.3. Data Collection

#### 2.3.1. MAPR GO and Optical Motion Capture System

This study was reviewed and approved by the Institutional Review Board at the Miami VA Medical Center and University of Miami. Data were simultaneously collected from five subjects using the Vicon Bonita OMCS (Vicon Motion Systems Ltd., Oxford, UK) and a 100 Hz MAPR sensor system. OMCS markers were placed on the lower limbs according to the lower limb Plug-in Gait marker system. Subjects walked 10 m with 2.5 m acceleration and deceleration zones appended to a 5 m capture zone. The capture zone was flagged via an Apple iPad Air 2 for all enrolled subjects using the custom mobile app. Each subject performed eight passes at self-selected walking speed (SSWS), as well as a static calibration trial for the Vicon system and an alignment trial consisting of 2.5 sit to stand motions and 10 steps for the MAPR system [3]. 

#### 2.3.2. IMU GO, CF, and EKF Algorithms

Six subjects were asked to perform four trials walking at SSWS across a 12.25 m walkway while instrumented with both a 50 Hz MAPR system and a 128 Hz Opal system, walking as described in [3]. The first and last two strides were discarded prior to analysis for extraction of constant velocity level ground walking. Data were recorded simultaneously via subjects instrumented with both Opal and MAPR systems. An alignment trial consisting of 2.5 sit to stand motions and 10 steps was also collected. 

### 2.4. Data Analysis

#### 2.4.1. MAPR GO and OMCS Knee Angular Velocity Analysis

**Figure 4:**
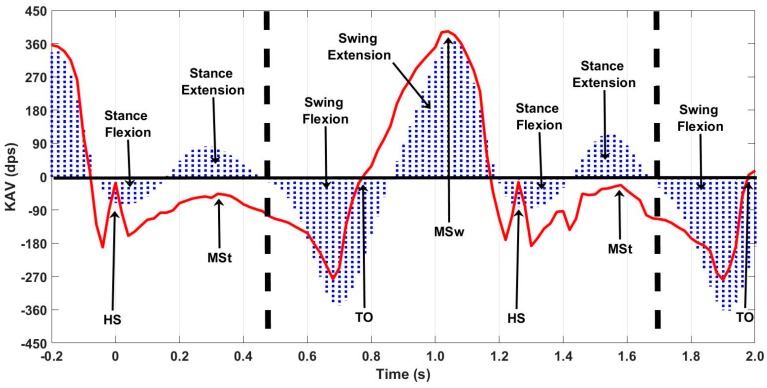
Swing and stance flexion intervals in the Vicon KAV. Zero-crossing in the Vicon KAV signal prior to swing flexion was used to delimit strides by the dashed lines. This zero-crossing was always found between MSt and TO. Blue area, Vicon KAV signal; Red line, MAPR SAV used to identify HS, MSt, and TO. HS, Heel-Strike; MSt, Mid-stance; TO, Toe-Off; MSw, Mid-swing; KAV, Knee Angular Velocity; dps, degrees per second.

**Figure 5:**
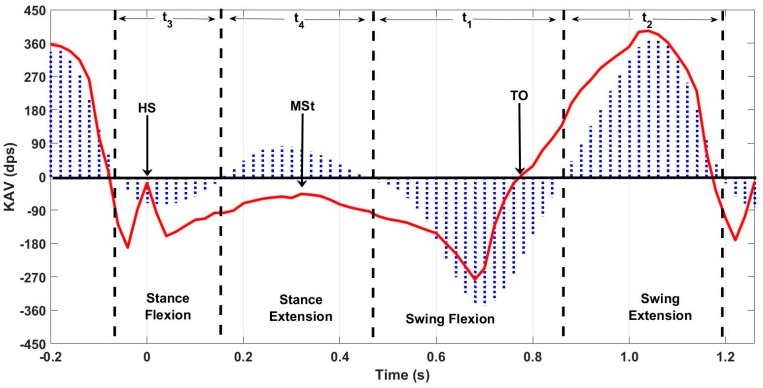
Swing and stance flexion time intervals. Times between zero-crossings in the Vicon KAV signal are delimited by the dashed lines and correspond to the physical time intervals of stance and swing flexion and extension. Blue area, Vicon KAV signal; Red line, MAPR SAV signal used to identify HS, MSt, and TO.

Vicon data were processed using Nexus 2.3 software to extract KAV signals for each limb. Then, data were manually segmented using the onset of knee flexion during stance as the delimiter of each stride. A zero-crossing detection algorithm was used to segment each stride into swing flexion, swing extension, stance flexion, and stance extension regions. The resultant KAV was low pass filtered using a 3rd order Butterworth filter with a 6 Hz cutoff. Heel-strike (HS), toe-off (TO), and mid-stance (MSt) were identified using the NZC algorithm on the MAPR aligned shank angular velocity (SAV) signal [3] (Figure 4). Characteristic features of the OMCS KAV and the MAPR KAV were manually analyzed to demonstrate whether the IMU KAV has an easily identifiable zero-crossing that exists between two reliably identifiable gait events. The gait cycle consisted of four reliably identifiable phases of knee angle: stance flexion, stance extension, swing flexion, and swing extension. Once segmented into these phases, peak angular velocity and interval timing were calculated for each flexion and extension period of stance and swing for each stride (Figure 5). Bland–Altman analysis was used to assess the mean difference and variation of the flexion and extension interval times between the MAPR and OMCS KAVs, as well as peak angular velocity within these intervals. Total gait cycle time (GCT) was calculated as time between subsequent onsets of swing flexion and a Bland–Altman analysis comparing GCT derived from the MAPR and OMCS KAVs was used as a measure of reliability of the identification of that point within the gait cycle. To quantify the integratory drift inherent in numerical integration of kinematic signals, displacement of the knee angle during flexion and extension were computed for stance, swing and stride intervals. Significance was assessed from zero mean using a *t*-test and root mean square error (RMSE) was reported for difference in displacement under each condition.

#### 2.4.2. Knee Angle Analysis

(1) MAPR GO and OMCS Knee Angle Analysis

Vicon data were processed using Nexus 2.3 software to extract knee angles for each limb. Bland–Altman analysis was performed to determine the difference in peak swing flexion between the MAPR GO algorithm and the integral of the OMCS KAV signal. Accuracy was measured as mean difference and precision as coefficient of variation. The average output per trial and per subject was compared. The integral of the OMCS KAV was chosen for comparison to remove the variable DC offset in the measured OMCS knee angle signal without distorting the knee angle curve.

(2) MAPR GO, CF, and EKF Knee Angle Analysis

The algorithm by Seel et al. was implemented for comparison, as described in the literature [8]. Data from gyroscopes and the accelerometers on the thigh and shank in the sensor reference frame were transformed into the body reference frame prior to calculation of knee angle using automatically detected rotation and transformation matrices. Each sensor type produced its own estimate of knee angle; the accelerometer estimate $\Psi_{a}$ and gyroscope estimate $\Psi_{g}$ were combined using a complimentary filter (Figure 2). A virtual alignment algorithm and a sensor distance minimization algorithm [7] was run on the alignment trial data to calculate an estimate of joint axis *j* and sensor distance from joint center *o* (Figure 2). Sensor distance from joint center *o* uses the assumption of the conventional gait model regarding the knee joint sensor to define distance from the sensor to the knee joint center in three dimensions. Convergence of the minimization algorithms was automatically verified and accomplished within fifty iterations. Additionally, an accelerometer offset algorithm was applied to zero out the accelerometer knee angle estimate during quiet standing when the knee angle is zero.

Peak swing flexion was calculated as displacement of the CF algorithm output relative to its closest neighboring trough to compensate for drift in the output signal. Knee angle estimates were collected under four conditions, representing each possible pairing of knee angle algorithm and IMU system: CF_Opal_, CF_MAPR_, GO_Opal_, and GO_MAPR_. A stride by stride analysis of each algorithm was performed within algorithms but across IMU systems (CF_Opal_ compared to CF_MAPR_, and GO_Opal_ compared to GO_MAPR_) to determine algorithm robustness to change in sensor type. A similar analysis was performed across algorithms but within systems (GO_Opal_ compared to CF_Opal_, and GO_MAPR_ compared to CF_MAPR_) to assess concurrent validity of the GO algorithm with the CF algorithm. RMSE was also calculated between algorithms within both the Opal and MAPR systems to quantify average difference between the GO and CF algorithms. Average CV of peak swing flexion was calculated for each IMU system/knee angle algorithm combination by averaging CV of peak swing flexion for each limb of each subject.

An Extended Kalman Filter (EKF) was used to calculate knee angle from the MAPR system (EKF_MAPR_) and peak swing flexion was calculated as trough to peak displacement. RMSE was calculated between the signals. EKF knee angle calculation was successful on five subjects. A stride by stride analysis was performed between the EKF_MAPR_ and GO_MAPR_ knee angle estimates comparing peak swing flexion using Bland–Altman analysis. 

## 3. Results

### 3.1. Knee Angular Velocity

**Figure 6:**
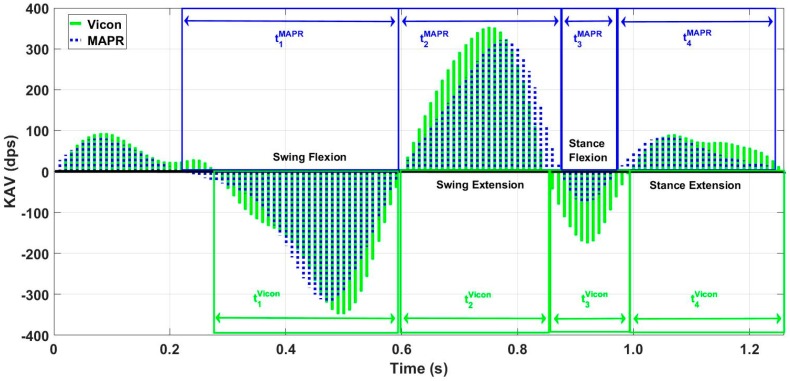
Time interval comparison of manually aligned Vicon and MAPR KAV Signals. While areas and signal profiles are similar, they differ in length of flexion and extension intervals. Boxes delimit the flexion and extension intervals of swing and stance determined by each KAV. Difference in length of time interval was used in Bland–Altman analysis. Blue, MAPR KAV signal; Green, Vicon KAV signal.

**Figure 7:**
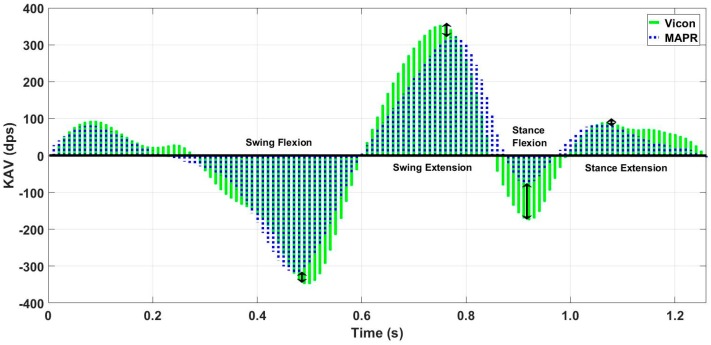
Peak angular velocity comparison of manually aligned Vicon and MAPR KAV Signals. While areas and signal profiles are similar, they differ in location of peaks and depth of peak near HS. Arrows show difference in peak angular velocity for each phase being analyzed using Bland–Altman. Blue, MAPR KAV signal; Green, Vicon KAV signal.

Overall, 40 passes were captured, leading to 80 potential knee angle trials when treating each limb as a separate entity. Visual analysis of the characteristic waveforms of the Vicon KAV and low pass filtered MAPR SAV confirmed that only four zero-crossings were seen per stride cycle and that the values corresponded to flexion and extension of the knee during gait (Figure 5). High accuracy and precision were found for the four-time intervals (t_1_, t_2_, t_3_, and t_4_) identified in the KAV signal when compared to the corresponding intervals in the Vicon signal using Bland–Altman analysis, where t_1_ is swing flexion, t_2_ is swing extension, t_3_ is stance flexion, and t_4_ is stance extension (Figure 6 and Table 1). The GCT also was zero mean with a low coefficient of variation (CV), showing that the point of inception of swing flexion is a repeatable measure of the gait cycle, which suggests that it is a stable point for resetting the knee angle. While mean deviation was similar for all four-time intervals, swing flexion and extension were three times less variable than stance flexion and extension. The KAV signal displayed a similar pattern, with CV 2–3 times higher for stance than swing. The MAPR signal persistently underestimated peak KAV during flexion when compared to the Vicon system by 20–60 dps (Figure 7 and Table 2). 

**Figure 8:**
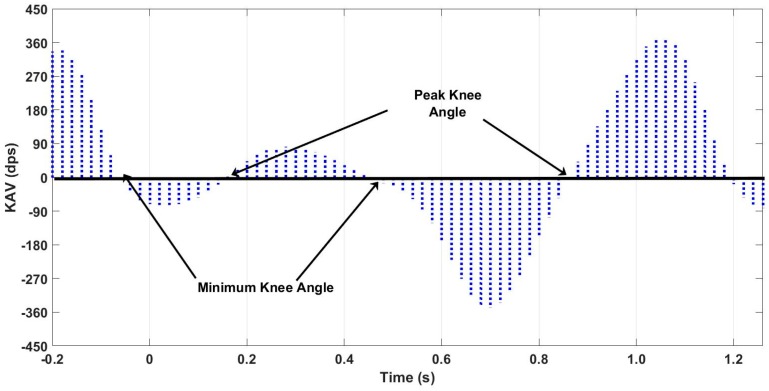
Peaks and troughs in the knee angular velocity signal and its relationship to knee angle.

Next, we compared the integrals of the MAPR and Vicon KAV signals to demonstrate that a single ZAU per stride is appropriate and sufficient for calculation of knee angle (Figure 6). Specifically, Vicon and MAPR KAVs were used to compare angular displacement calculated by both systems. The KAV waveform observed by the Vicon and MAPR systems share similar characteristics. The existence of a fixed number of zero-crossings in the KAV of the MAPR and the agreement of the MAPR KAV flexion and extension intervals with the Vicon KAV derived intervals demonstrates that the chosen algorithm characteristics were appropriate (Figure 8). The beginning of swing flexion, represented as the black dashed lines in Figure 4, always began after MSt but before TO.

**Figure 9:**
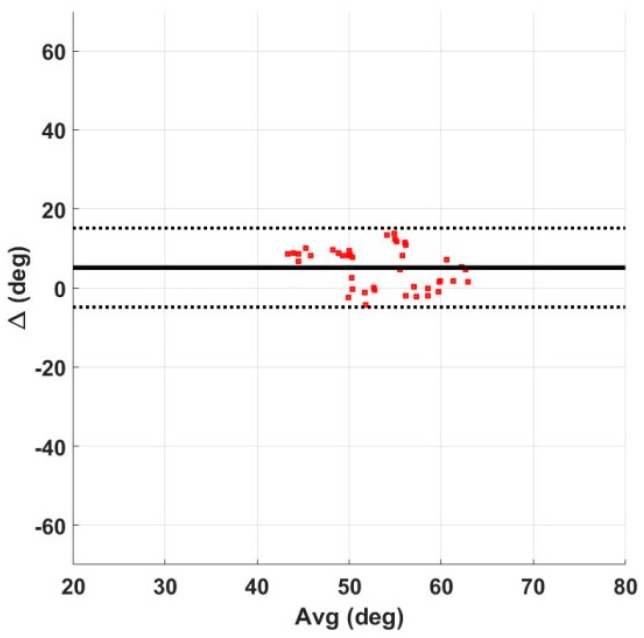
Comparison of GO algorithm with Vicon for peak swing flexion.

While peak angular velocity during each of the swing and stance flexion and extension periods was underestimated by the MAPR system (Figure 7), the relative location of zero-crossings, which represent transitions between flexion and extension of the knee, is highly accurate with a mean difference of less than 15 ms (Table 1). Since the KAV signal is smooth and continuous, the Fundamental Theorem of Calculus necessitates that zero-crossings in the KAV signal necessarily correspond to minima and maxima of the knee angle. The positive to negative zero-crossings in the KAV correspond to minima in the signal and represent points within the gait cycle where the integral can potentially be reset (Figure 9). Agreement between the MAPR and Vicon systems for KAV interval timings demonstrates that the two signals are observing the same minima and maxima. Upon inspection of the MAPR KAV and SAV signals, the KAV zero-crossing corresponding to the first minimum knee angle point always occurs between MSw and HS, while the KAV zero-crossing corresponding to the second minimum knee angle point always occurs between MSt and TO. This corresponds to the known motion of the knee joint during the gait phase. Following HS, the knee flexes to accept the weight of the body as stance begins. After MSt and before TO, the knee flexes to prepare for foot clearance during swing limb advancement. Thus, two zero-crossings exist in the KAV signal that correspond to minima in the knee angle signal and can be reliably identified using gait events detected in the SAV signal.

The MAPR system showed 1.5–3 times the variability of the Vicon system (Table 3). A *t*-test showed that the integral of the MAPR KAV over an entire gait cycle was significantly different from zero, while the average displacement over the stance and swing components were not. Integratory drift over a single stride is typically within 2.67° ± 3.72° (Table 3).

Average relative displacements over both the swing and stance motions of the knee individually were not significantly different from zero (Table 3), in agreement with the observations of the Vicon system. Net change in the knee angle over an entire stride should be, on average, zero. The OMCS system yielded zero mean displacement over stance and swing intervals individually, as well as the entire stride. This suggests that in healthy populations, knee angle after each gait phase should approach zero as the knee extends after each peak flexion. The significant difference of the integral of the MAPR KAV over the entire stride, however, shows that an average of 2.67° per stride of drift accumulates in the signal after a single stride (Table 3). While this amount of drift is within the 5° error accepted as a clinical standard for minimum detectable change [17], it increases above this threshold after accumulation over more than a single stride. Therefore, this demonstrates that the MAPR KAV integral needs to be reset once per stride to keep the knee angle error due to drift within appropriate levels. Since the integral of the Vicon KAV was not significantly different from zero mean for stance, swing, or the entire stride, both zero-crossings in the MAPR KAV that correspond to minima in the knee angle signal can be used as ZAU points. The knee angle drifts by an acceptable 2.67° per stride, meaning that the integral can be reset once per stride.

### 3.2. Knee Angle Validation 

#### 3.2.1. Agreement between the GO Algorithm and OMCS

**Figure 10:**
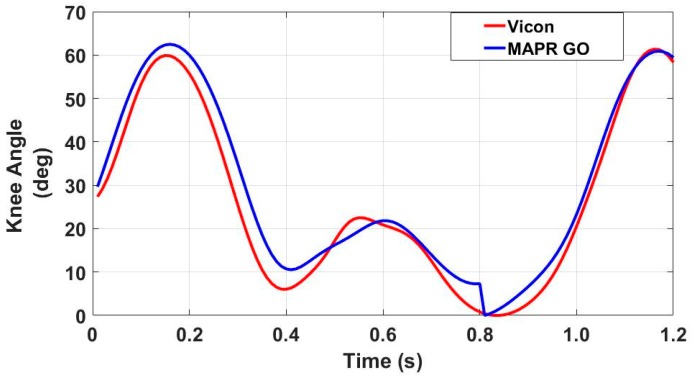
Representative knee angle waveform extracted from a single pass using a motion capture system and IMU-based gyroscope only algorithm. Similar waveforms were observed between the two signals, with comparable peak heights and the largest difference in the signal set in a trough in between peaks.

Performance of the GO algorithm was comparable to OMCS data, with the GO either underestimating or accurately estimating the knee angle. A mean difference of approximately 5° was found when comparing the GO output for peak swing flexion with that of the Vicon system (Table 4). The coefficient of variation of the Bland–Altman analysis was approximately 10%. The GO algorithm underestimated peak swing flexion by an average of 5° when compared to the Vicon system. Data were clustered, with several zero-mean trials along with several trials that were off by approximately 10° (Figure 9). Shape of the waveform was similar between the Vicon and GO algorithm (Figure 10), with a small discontinuity in the GO algorithm signal at the zero knee angle (ZKA) update.

#### 3.2.2. Agreement between the GO Algorithm and IMU Algorithms

All test subjects were healthy adults without gait deviations. A total of 538 strides were analyzed for SSWS and 426 strides were analyzed for the fast walking speed. Bland–Altman analysis comparing both GO and CF algorithm performance across IMU data acquisition systems (Table 5) showed that the mean peak swing flexion estimate did not vary across IMU systems, but the 95% CI was more than ±5°. However, the variance of the GO algorithm across systems was several degrees less than that of the CF algorithm. Bland–Altman analysis comparing the output of each algorithm across IMU systems showed that the GO algorithm consistently overestimated knee angle compared to the CF algorithm by an average of 5° across individual steps, trial averages, and subject averages (Table 6). Average RMSE of GO and CF was roughly 6.5° for both the Opal and MAPR systems (Table 7). All four combinations of IMU system and knee angle algorithm yielded low average CV that were less than 6% (Table 8). Bland–Altman analysis comparing both GO and EKF algorithm performance with the MAPR system yielded similar results as the GO and CF algorithm comparison (Table 5). There was a smaller mean difference between the GO and EKF algorithms than the GO and CF algorithms with a slightly larger CV. RMSE analysis showed a smaller RMSE between the GO and EKF algorithms than the GO and CF algorithms (Table 7). 

**Figure 11:**
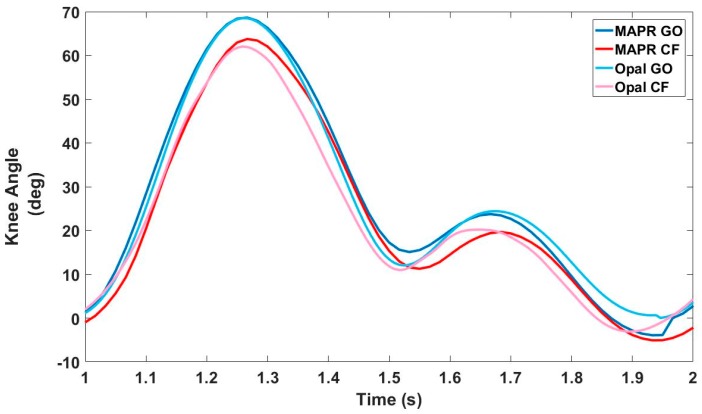
Representative knee angle waveform under each condition. GO and CF performance were distinct from each other. GO consistently overestimated knee angle compared to CF.

A representative waveform for each of the algorithms calculated from each IMU system is presented in Figure 11. By the end of the stride, the CF produced a knee angle estimate that is several degrees negative, representing overextension of the knee. This occurred across IMU systems and is most likely due to integratory drift. Because the GO algorithm resets the knee angle to zero prior to the next stride, the estimated knee angle output by the GO algorithm is higher than the CF algorithm across IMU systems as well. The degree and sign of drift varied across subjects, suggesting that drift was partially a function of how the individual subject walked. 

## 4. Discussion

The results demonstrate that the difference between the GO algorithm and the three criterion measure systems is acceptable for mobile clinical applications which require continuous monitoring. Currently, there is no standard clinical system for continuous knee angle measurement with mobile health devices. Assessment of decreased knee angle flexion during stance or swing is obtained via observational gait analysis by the clinician. This method, however, cannot be used outside the clinic when the patient is unsupervised, nor does it provide objective quantification while within the clinic. As can be seen in Table 8, the GO algorithm output stable estimates of peak swing flexion that were consistent within the subject, making it an improvement over current clinical standards. While the direct integration of the gyroscope data underestimated peak knee angle swing flexion when compared to an OMCS system (Table 4), it overestimated compared to both the validated CF IMU algorithm and the EKF. The RMSE of the GO algorithm compared to the three criterion measures was within 5–7° (Table 6). Likewise, the underlying swing and stance flexion and extension periods in the KAV observed by both the MAPR and OMCS KAV signals were concurrent with high accuracy and precision (Table 1), validating the physical underpinnings of the GO algorithm. While the integral over the entire stride of the MAPR KAV did have an average drift of 2.67° (Table 3), this is acceptable error as long as the integral is reset every stride. The overall waveform of the MAPR KAV and knee angle were comparable to that of the OMCS KAV (Figure 6 and Figure 7) and knee angle (Figure 10). This implies that the estimates of GO algorithm are on average an accurate representation of knee angle compared to the output of the two reference systems. 

### 4.1. GO Algorithm Concurrence with Reference Systems

Both the GO and CF algorithms behaved similarly across and within IMU systems. While the mean difference for each algorithm across IMU systems was close to zero, the CV was relatively high, with the CF algorithm being more variable across IMU systems than the GO algorithm. Since the Opal system was attached via elastic straps on top of the MAPR system, stride dependent noise due to sensor motion would be higher in the Opal system than in the MAPR system and could have led to variability in comparisons between the two systems but within algorithms (Table 5). Restrictions in attachment of the two systems prohibited interchange of positions to act as a control. Secondly, high noise levels at initial heel contact appear in the filter estimate through both the accelerometer and gyroscope data despite the implementation of filtering techniques described by Seel et al. This is supported by the higher CV of the between algorithm comparison seen in the Opal system than the MAPR system (Table 8). Increased variability for the CF algorithm can therefore be attributed to the dependence of this algorithm on accelerometer data and its sensitivity to vibratory gait phases.

While OMCS and IMU systems purport to provide absolute measures of knee angle, each operates with underlying assumptions of the biomechanical behavior of the knee joint that limit their accuracy. IMU systems suffer from sensor drift due to changes in the underlying tissue to which they are affixed over the course of the gait cycle. Both IMU and OMCS abstract kinematic parameters, project them onto a biomechanical model, and extract an angle based on the assumption that a single joint accurately represents the dynamics of a double joint moving in three dimensions. Inter-session and inter-rater reliability of OMCS is often a confounder that remains unconsidered when evaluating reliability and accuracy of gait analysis systems [17]. While the clinical threshold for minimum detectable change is 5° [17], intersession and interrater confounders can increase the MDC and decrease ICC of characteristic values in the knee angle signal. A minimum detectable change in peak swing flexion of 7.33° and 6.39° was found under intrarater-intersession and interrater-intersession comparisons, respectively, with ICCs of 0.57 and 0.62 [17]. A systematic review of the reliability of gait measurements using optical motion capture systems demonstrated an average 4° of within system error [18]. Choice of marker set has also been shown to significantly influence gait parameter output [19]. Similarly, a research oriented, commercially available, full body 16 IMU system found an average error of 9.5° ± 7.0° for knee flexion and extension when compared to an optical motion capture system, but high reliability within the system [20]. This study concluded that the error between the systems was due to marker placement error. Similarly, while many studies have been able to obtain high degrees of concurrence between IMU and optical motion capture systems for calculation of knee angle, many of these consist of single subject studies or calculate knee angle as the difference in angle between markers placed on the IMUs instead of the body [8]. 

In this study, both reference systems suffered from variations in peak height due to drift and offset, and the Vicon system was also only able to collect a limited number of steps. Although the CF algorithm is supposed to be able to completely remove drift due to numerical integration of the KAV signal, subject based variance resulting in minimum knee angles of up to −10° (10° of overextension) were seen using the CF algorithm and the EKF. This most likely was due to high levels of noise obfuscating the knee stance flexion peak in the KAV and shifting the knee angle resultant signal down. The EKF did not produce a viable knee angle signal for one of the six subjects using the MAPR system, most likely due to noise in the accelerometer signal and sensor drift. Similarly, overextension and drift in the knee angle was prevalent in the Vicon data as well. Although drift compensation was performed prior to analysis by subtracting the value of the closest neighboring minimum for CF and by integration of the KAV for the Vicon analysis, it was unable to completely remove drift from the signal prior to comparison with GO. This argument is supported by the larger size of the RMSE for most subjects when compared to the mean difference of peak swing flexion seen in the Bland–Altman analysis (Table 7). The RMSE between algorithms being higher than their mean difference at peak swing flexion implies that there was a larger discrepancy between the two signals after peak swing flexion than before it. In conjunction with the visible drift in the shank angular velocity signal, this demonstrates the strength of the GO algorithm in accounting for drift within acceptable limits. 

In light of these findings, a mean difference of 5° for peak knee angle at swing flexion (Table 4) and the corresponding differences in peak knee angular velocities during stance and swing are within the measurement variability of the Vicon system. The advantage of the GO algorithm over the OMCS system is that it replaces manual measurements and calibrations that are prone to interrater and intersession error and varying degrees of performance with standardized mathematical models, replacing the multiple rater problem with a single rater (the IMU system). Since the OMCS KAV was derived from the knee angle, errors in the peaks of the knee angle would necessarily propagate into the KAV. Zero-crossings of the KAV, representing the overall direction of motion of the markers in space, showed high accuracy between the two systems. Since they represent the direction of motion of the markers in space relative to each other and not the magnitude of that motion, they ought to be more robust to the inherent variability of the OMCS. The zero-crossing prior to swing flexion (t_swf_) is able to be identified with high reproducibility and the total integral of the knee angular displacement in the Vicon system between subsequent zero-crossings at t_swf_ is on average zero.

### 4.2. Advantages of the GO Algorithm 

The GO algorithm has been shown to be an appropriate method for knee angle calculation that relies on a ZAU to eliminate drift and calculates knee angle with only a single sensor type. Unlike algorithms such as the EKF that operate via a 3DGA paradigm, the GO algorithm does not depend on complicated statistical techniques for determination of sensor orientation. In addition, it has been validated at low sampling rates that may be inappropriate for use of common orientation algorithms due to the non-linearity of the motions they track. While the CF algorithm [8] presents a novel method for knee angle calculation, it still requires the use of accelerometer data, which is disadvantageous for application in mobile devices for continuous monitoring applications for several reasons. First, accelerometer data yield more accurate estimates of sensor orientation under a sample–decimate–transmit paradigm because of its relatively large Gaussian noise component. Second, acceleration is susceptible to vibratory modes [21] that can be a function of subject anatomy and variable based on subject driven donning of the sensor system. Finally, accelerometer data increase bandwidth requirements, curtailing sampling rate and decreasing battery life in mobile systems [22].

While the GO algorithm may be inappropriate for subject populations that never fully extend their knee, it is appropriate for amputee and cerebral palsy populations that exhibit decreased swing flexion during gait. The original CF algorithm was validated in an amputee population [8]. While the KAV signal should show a region of zero angular velocity in subject populations that do not bend their knee at stance, the shank angular velocity signal should still contain the same characteristic peaks the correspond to MSt and TO. Thus, the ZKA point prior to swing flexion should still be identifiable in the signal. Similarly, for populations that do not fully extend the knee at the end of stance flexion, the KAV analysis would propose that a knee angle reset at the other knee angle minimum may be an appropriate alternate to the ZAU employed in this implementation of the GO. In such a situation, a state machine of some form would decide which ZAU to employ and the subject’s knee angle would be tracked using that method. Since only four zero-crossings were observed per stride, knowledge of gait events identified in the shank IMU signal could be employed to reliably identify the other zero-crossing corresponding to a knee angle minimum. Further studies should investigate the ability of the minimum change that is detectable by this algorithm in pathologic populations. Likewise, the GO should be compared to standard clinical practices for identification of decreased knee angle using observational gait analysis. Bias estimates may be able to be computed and used to remove drift without completely resetting knee angle every stride. Unfortunately, such an analysis requires the collection of more consecutive strides than is accessible within the constraints of a gait lab, so additional study design problems will have to be overcome to investigate the accuracy of such a method. 

## 5. Conclusions

The novel GO algorithm can calculate knee angle using only angular velocity data and gait phase information obtained from IMUs attached above and below the knee joint. The peak swing flexion estimated by the GO algorithm was between that of the three criterion measures (an established complementary filter IMU algorithm, an Extended Kalman Filter IMU algorithm and optical motion capture system) with a mean difference of ≤5° from any system. The GO algorithm was more effective in accounting for bias and integratory drift than other measures. The low average coefficient of variation (<5%) of the GO algorithm demonstrates that it produces stable estimates of peak knee flexion, which are needed for continuous monitoring applications. Since the GO algorithm performed well under sampling rate and bandwidth restrictions of a mHealth system, it provides a potential solution for objective measurement of knee angle in the home/community for tele-rehabilitation applications. A potential application is for lower limb amputees, who demonstrate decreased knee flexion during walking and have a high risk for falls. Future work would include combining the GO algorithm with other algorithms for continuous monitoring of gait and providing biofeedback for reducing gait abnormalities. 

## Figures and Tables

**Table 1:** Bland–Altman Analysis Comparing Interval Times of MAPR and OMCS KAV during Flexion and Extension in Stance and Swing.

		Time (s)		
		Mean	LoA	CV
Stance	Flexion (t_3_)	0.013	[−0.043, 0.068]	17%
	Extension (t_4_)	0.01	[−0.075, 0.095]	17%
Swing	Flexion (t_1_)	−0.013	[−0.055, 0.029]	6%
	Extension (t_2_)	−0.015	[−0.043, 0.014]	5.3%
	GCT	−0.0049	[−0.052, 0.043]	2.3%

LoA, Limits of Agreement; CV, Coefficient of Variation; GCT, Gait Cycle Time.

**Table 2:** Bland–Altman Comparing Peak Values of MAPR and OMCS KAV during Flexion and Extension in Stance and Swing.

		Peak Angular Velocity (Deg/s)		
		Mean	LoA	CV
Stance	Flexion (t_3_)	51	[−1.4, 103]	27%
	Extension (t_4_)	19	[−5, 43]	16%
Swing	Flexion (t_1_)	61	[−11, 130]	12%
	Extension (t_2_)	41	[−16, 98]	9.6%

LoA, Limits of Agreement; CV, Coefficient of Variation; GCT, Gait Cycle Time.

**Table 3:** Average value of angular displacement obtained by integrating knee angular velocity for inertial measurement units and optical motion capture systems. * significant difference.

	MAPR		Vicon	
	Mean ± SD (Deg)	*p*-Value	Mean ± SD (Deg)	*p*-Value
Swing	0.795 ± 4.22	0.3283	−0.12 ± 1.39	0.9005
Stance	−1.88 ± 4.93	0.0536	−0.065 ± 2.72	0.6494
Stride	2.67 ± 3.72 *	<0.001	−0.56 ± 2.78	0.9158

**Table 4:** Comparison of GO Algorithm with Vicon KAV Integral. Bland–Altman analysis of peak swing flexion detected from GO and Vicon.

	Angular Displacement (Deg)		
	Mean	LoA	CV
Trial	5.2	[−4.8, 15]	9.6%
Subject	5.0	[−5.3, 15]	9.9%

LoA, Limits of Agreement; CV, Coefficient of Variation.

**Table 5:** Bland–Altman analysis of peak swing flexion within algorithms across IMU systems.

	Level of Analysis	Peak Swing Flexion (Deg)		
		Mean	LoA	CV
CF_MAPR_ vs. CF_Opal_	Stride	−0.66	[−11, 9.5]	9.6%
	Trial	−0.56	[−10, 8.9]	9%
	Subject	−0.065	[−9.9, 8.6]	8.9%
GO_MAPR_ vs. GO_Opal_	Stride	−1.3	[−9.3, 6.7]	6.9%
	Trial	−1.1	[−8.4, 6.2]	6.3%
	Subject	−1.1	[−8, 5.9]	6.0%

GO_MAPR_, GO algorithm knee angle estimate using MAPR data; GO_Opal_, GO algorithm knee angle estimate using Opal data; CF_MAPR_, CF algorithm knee angle estimate using MAPR data; CF_Opal_, CF algorithm knee angle estimate using Opal data.

**Table 6:** Bland–Altman analysis of peak swing flexion within algorithms across IMU Systems.

	Level of Analysis	Peak Swing Flexion (Deg)		
		Mean	LoA	CV
GO_MAPR_ vs. CF_MAPR_	Stride	−5.5	[−14, 2.5]	7.2%
	Trial	−5.5	[−12, 0.81]	5.7%
	Subject	−5.4	[−11, 0.54]	5.4%
GO_Opal_ vs. CF_Opal_	Stride	−4.9	[−12, 2]	6.3%
	Trial	−5.0	[−11, 1.4]	5.8%
	Subject	−5.0	[−11, 1.1]	5.6%
GO_MAPR_ vs. EKF_MAPR_	Stride	−2.4	[−11, 6]	7.4%
	Trial	−2.7	[−11, 5.3]	7.0%
	Subject	−3.6	[−10, 3.1]	6.6%

GO_MAPR_, GO algorithm knee angle estimate using MAPR data; GO_Opal_, GO algorithm knee angle estimate using Opal data; CF_MAPR_, CF algorithm knee angle estimate using MAPR data; CF_Opal_, CF algorithm knee angle estimate using Opal data; EKF_MAPR_, Extended Kalman Filter knee angle estimate using MAPR data.

**Table 7:** RMSE (deg) between CF and GO for each subject.

	Subject						
	1	2	3	4	5	6	Mean
GO_MAPR_ vs. CF_MAPR_	4.45	6.50	5.33	9.60	7.53	6.88	6.72
GO_Opal_ vs. CF_Opal_	10.99	5.74	7.57	4.13	6.29	5.16	6.32
GO_MAPR_ vs. EKF_MAPR_	4.37	6.77	6.45	4.39	6.42	-	5.68

GO_MAPR_, GO algorithm knee angle estimate using MAPR data; GO_Opal_, GO algorithm knee angle estimate using Opal data; CF_MAPR_, CF algorithm knee angle estimate using MAPR data; CF_Opal_, CF algorithm knee angle estimate using Opal data; EKF_MAPR_, Extended Kalman Filter knee angle estimate using MAPR data; RMSE, Root Mean Square Error.

**Table 8:** CV (%) for peak swing flexion across IMU systems and knee angle algorithms.

	MAPR	Opal
GO	4.48	3.79
CF	5.13	4.26

MAPR, Multi-Axial Profile Recorder; CF, Complimentary Filter Algorithm; GO, Gyroscope Only Algorithm; CV, Coefficient of Variation.
